# Development of Peptide-Based Vaccines for Cancer

**DOI:** 10.1155/2022/9749363

**Published:** 2022-03-15

**Authors:** Noraini Abd-Aziz, Chit Laa Poh

**Affiliations:** Centre for Virus and Vaccine Research (CVVR), School of Medical and Life Sciences, Sunway University, 47500 Subang Jaya, Selangor, Malaysia

## Abstract

Peptides cancer vaccines are designed based on the epitope peptides that can elicit humoral and cellular immune responses targeting tumor-associated antigens (TAAs) or tumor-specific antigens (TSAs). In order to develop a clinically safe and more effective vaccine for the future, several issues need to be addressed, and these include the selection of optimal antigen targets, adjuvants, and immunization regimens. Another emerging approach involves the use of personalized peptide-based vaccines based on neoantigens to enhance antitumor response. Rationally designed combinatorial therapy is currently being investigated with chemotherapeutic drugs or immune checkpoint inhibitor therapies to improve the efficacy. This review discusses an overview of the development of peptide-based vaccines, the role of adjuvants, and the delivery systems for peptide vaccines as well as combinatorial therapy as potential anticancer strategies.

## 1. Introduction

Cancer ranks as the second leading cause of death in the world, with 10 million deaths being reported in 2020 [1]. Although cancer survival rates have increased dramatically over the years in most countries, more research studies are needed to enhance the survival of patients suffering from this deadly disease. Immune evasion has been identified to be one of the defining aspects of cancer cells, contributing to the emergence of immunotherapies that enhance the host immune system to recognize and eliminate cancer cells. Recently, immunotherapeutic approaches such as cytokine therapy, immune checkpoint inhibitors, monoclonal antibody-based therapies, chimeric antigen receptor (CAR) T cells, and cancer vaccines have significantly improved the clinical outcome of cancer treatments. Cancer vaccines work by instructing the immune system to recognize tumor antigens as foreign [2]. It can be used prophylactically to prevent tumor development or therapeutically to treat patients who have already been diagnosed with cancer [3].

There are two types of prophylactic cancer vaccines approved by the Food and Drug Administration (FDA) which are linked to two cancer-causing viruses and have been shown to be effective in lowering the global burden of human papillomavirus (HPV) and hepatitis B virus (HBV) (Table 1). There are several HBV vaccines marketed under commercial names such as Engerix-B, Recombivax HB, and Heplisav-B. Engerix-B, Recombivax HB, and Heplisav-B are viral-like particles (VLPs) utilizing hepatitis B surface antigens produced in yeast. Gardasil and Cervarix, on the other hand, are generated from VLPs consisting of the single capsid protein L1, which provides protection against HPV types 16 and 18 [4]. The effectiveness of these vaccines is dependent on the production of a robust neutralizing antibody response against immunodominant viral antigens. Cervarix is a bivalent vaccine made up of proteins from HPV-16 and HPV-18, while Gardasil is a quadrivalent vaccine made up of VLPs from HPV-6, HPV-11, HPV-16, and HPV-18 [5]. The FDA has also approved the use of Gardasil-9, a 9-valent HPV vaccine for females and males from 9 to 45 years of age. The vaccines have outstanding safety and immunogenicity profiles and have provided long-term protection against persistent infections in vaccinated women [6].

Therapeutic cancer vaccines differ from prophylactic vaccines as they induce an immune response to an existing tumor or cancer cells that have survived earlier therapies [7]. Depending upon the type of cancer, an individual might have the primary tumor surgically removed but leave behind a small cancer mass. Immunotherapy entails essential steps of immune response to an antigen, activation of cancer-specific effector and cytotoxic T cells that are trafficked into tumors, recognition of cancer cells, and killing of tumor cells. To date, there are three FDA-approved therapeutic cancer vaccines, and these are Bacillus Calmette-Guerin (BCG), sipuleucel-T and talimogene laherparepvec (T-VEC) [8] (Table 1). BCG derived from *Mycobacterium bovis* has been used to treat the nonmuscle invasive bladder cancer subtype. The approval was based on a phase III trial that demonstrated a five-year disease-free survival of 45% with BCG use [9]. Sipuleucel-T (Provenge) is another FDA-approved therapeutic vaccine for prostate cancer patients. This vaccine employs the immune cells of patients such as dendritic cells, B and T cells, and natural killer cells isolated through leukapheresis to stimulate an immune response against prostatic acid phosphatase (PAP) [10]. The vaccine approval was based on the success of a placebo-control study involving 512 patients randomly assigned to receive sipuleucel-T or placebo in a 2 : 1 ratio [11]. T-VEC, a genetically modified oncolytic virotherapy derived from herpes simplex virus type I (HSV-1) encoding the human granulocyte-macrophage colony-stimulating factor (GM-CSF), is the most recent FDA-approved vaccine for the treatment of unresectable cutaneous, subcutaneous, and recurrent nodal lesions in patients with melanoma following initial surgery. In a phase III trial, patients with unresected stage IIIB to stage IV melanoma who were injected with T-VEC had a greater durable response rate (16.3%) compared to those who were injected with GM-CSF (2.1%) [12].

Therapeutic cancer vaccines including peptide, DNA, RNA, protein, and tumor cell-based vaccines aim to produce new or enhance existing tumor-specific T cell responses against tumor cells [13–15]. Peptide-based vaccine is a vaccination approach with minimal side effects that use synthetic tumor-associated or specific peptides or a combination of peptides, designed to elicit peptide-specific T cells. These peptides are presented on the surface of human leukocyte antigen (HLA) molecules for recognition by T cell receptors of CD4^+^ and CD8^+^ T cells. In this review, we discuss the selection of target antigens, mechanism of peptide-based vaccines, delivery methods and role of adjuvants, clinical applications, and combinations with other immunotherapies for cancer treatment.

## 2. Tumor Antigens

The selection of tumor antigens is of utmost importance to develop a cancer vaccine. The ideal antigen should only be expressed by cancer cells and is highly immunogenic. The chosen antigen should also be present on all cancer cells which play a crucial role in cancer cell survival and protect against immune escape by mutations or loss of antigens in tumor cells. Tumor antigens can be classified as tumor-associated antigens (TAAs) or tumor-specific antigens (TSAs). Different types of tumor antigens are given in Table 2.

TAAs are self-antigens that are exclusively expressed in tumor cells, but have low levels of expression in normal cells. TAAs are categorized into three main groups such as overexpressed antigens, differentiation antigen, and cancer-testis antigens. The overexpressed antigens are produced at a higher level in tumor cells when compared to normal cells, and many antigens fall within this group such as HER-2, hTERT, MUC-1, mesothelin, and p53 [16–19]. Differentiation antigens are normally not expressed in adult tissues, and they include gp100, MART-1, prostate-specific antigen (PSA), prostatic acid phosphatase (PAP), and tyrosinase [20–24]. Another group of TAA is the cancer-testis antigens which are solely expressed in immune-privileged sites and can avoid immune recognition (e.g., MAGE-A1, MAGE-A3, NY-ESO-1, and PRAME) [25–28]. There are several challenges linked to the development of vaccines against TAAs. Since TAAs are self-antigens, both B and T cells that recognize these antigens are typically eliminated by central and peripheral tolerance and are inefficient in eliciting immune responses. Thus, cancer vaccines using TAAs must be powerful enough to “break” the tolerance mechanisms [20]. The disadvantage of utilizing TAAs in cancer immunotherapy is the risk of inducing autoimmunity as these antigens are also expressed in normal tissues and are commonly characterized by low immunogenicity, and T cells with low-affinity receptors are unable to mediate effective antitumor response. The use of effective adjuvants and costimulator in the formulation, as well as repeated vaccinations, could solve the problem of boosting the immunogenicity of the antigens and yielding clinical benefits for cancer patients [34].

Another notable cancer antigen is the tumor-specific antigen (TSA) or neoantigen. TSAs include antigens from oncoviruses and neoantigens encoded by mutated cancer genes [35]. The oncogenic viral antigens are expressed in cells infected with viruses that have subsequently experienced cellular transformation. Oncogenic viral antigens have been targeted by both prophylactic vaccines, such as HPV or HBV, and also in therapeutic vaccines to treat existing tumors. The most common targeted oncogenic viral antigens are Epstein–Barr virus (EBV) latent membrane proteins (LMP-1 and LMP-2), HPV-E6/E7, and HTLV-1 [29–32]. However, most tumors develop as a result of genetic instability and various mutations including translocations, frame-shift, or point mutations, which could lead to the production of new proteins, truncated proteins, or exposure of previously hidden epitopes that were distinct from the normal self-proteins. There are few mutated antigens that have been discovered where the mutated peptide is shared across cancer types, and the most common reported shared mutations were KRAS, NRAS, BCR-ABL translocation, ETV6, NPM/ALK, and ALK [33].

Neoantigens are highly immunogenic as they harbor mutations, can escape immune tolerance, and are recognized as nonself by the immune cells [36]. In addition, targeting TSAs should be less likely to induce autoimmunity. While neoantigens have long been known as attractive tumor targets, huge identification of these antigens has only recently become feasible due to the availability and lower cost of next-generation sequencing [37]. Developing a cancer vaccine that targets a patient's specific neoantigens necessitates a tailored strategy including sequencing the patient's tumor genome, identifying the mutations, predicting the neoantigens using computerized algorithms, and constructing a vaccine that expresses the predicted neoantigens [35]. Neoantigens have become ideal targets for successful immune responses as a result of the following efforts: tumor mutation burden and neoantigen load were associated with stronger antitumor T cell responses and improved clinical outcome [38], the number of neoantigen-specific T cells was shown to be higher in cancer patients who reacted to immune checkpoint inhibitors and other immunotherapies [39], and direct in vivo cytotoxicity by neoantigen-specific T cells was reported in various mouse tumor models [40]. The concept of eliciting an immune response against neoantigens is a potential “personalized medicine” strategy, but its clinical translation required a multistep process and could be challenging [41]. Eventhough personalized peptide-based cancer vaccines are in their infancy, the advent of whole exome sequencing (WES) and single-cell RNA sequencing (scRNA-Seq) enables the quick detection of nonsynonymous mutations which result in neoantigens and also allows HLA allele genotyping.

## 3. Peptide-Based Cancer Vaccines

A cancer vaccine aims to activate the immune system of patients, priming it to recognize, attack, and destroy tumor cells. Peptide vaccination approaches are being employed to design personalized vaccines based on tumor-specific antigens using synthetic peptides. Peptide-based cancer vaccines are generally made up of 20–30 amino acids that contain specific epitopes from antigens known to be highly immunogenic to elicit the desired immune response. Peptide vaccine has several advantages over other types of vaccines, particularly concerning safety and ease of production. Aside from their safety which was reported in many trials, peptide vaccines have been shown to induce T cell responses. However, challenges still remain to improve their immunogenicity. To be efficacious, peptide-based cancer vaccines must have CD8^+^ T cell epitopes to activate cytotoxic T lymphocytes (CTL) antitumor immunity via the antigen cross-presentation pathway, as well as CD4^+^ T cell epitopes to activate T helper cells, which sustain CTL effector functions [42]. Thus, the sequence length of the peptide vaccine is critical for promoting a robust immunogenic response.

Peptide vaccination generally employs two types of peptides. Short peptides, typically 8–12 amino acids, tend to have a short half-life and are easily degraded in serum. They bind to HLA class I groove on the surface of nucleated cells even without further processing by professional antigen-presenting cells (APCs). However, this could result in tolerance or short-term induction of CD8^+^ T cells, without concurrent activation of CD4^+^ helper T cells [43, 44]. Shorter peptides are generally HLA-type restricted as their lengths do not allow for the diversity necessary for the high polymorphisms of HLA in the general population [45, 46]. They are commonly conjugated to a carrier protein, allowing them to be taken up and processed by APCs to elicit an effective immune response. Synthetic long peptides (SLPs) which are usually 20 amino acids or more are more stable and immunogenic than short peptides as they are taken up and processed by APCs to produce peptides to be presented by both HLA class I and II molecules, leading to the induction of strong and long-lasting antitumoral immune responses involving CD4^+^ T cells, CD8^+^ T cells, and antibody production by B cells [43, 44]. SLPs have successfully been demonstrated to induce a robust immunogenic response, and they are shown to be more immunogenic than the whole antigen from which they were derived [47].

The peptide-based vaccine in clinical trials commonly carried multiple epitopes against multiple targets, as opposed to in vitro studies which generally rely on a single peptide. Targeting multiple epitopes derived from different antigens simultaneously could overcome tumor immune escape via antigen loss. The combination of MHC class I and class II epitopes was reported to elicit a balanced induction of CD4^+^ and CD8^+^ effector T cell activation, which led to the persistence and survival of effector cells in vivo [48]. Thus, these multiepitope peptide-based vaccines have been shown to have clinical benefits against tumors and are well tolerated. Rabu et al. had designed SLPs generated from MELOE-1 melanoma antigen comprising class I and class II epitopes separated by an artificial cathepsin-sensitive linker (LLSVGG), which led to a major impact on the presentation of the CD4^+^ and CD8^+^ T cell epitopes [49]. Cathepsin is an essential protease in dendritic cells (DC) involved in antigen presentation [50, 51]. Utilization of optimal cathepsin protease-sensitive linker sequence had significantly enhanced cross-presentation to CD8^+^ T cells, and vaccination with such SLPs was shown to reduce tumor growth in vivo [49]. The findings showed that SLPs could be used in future cancer vaccination trials to potentially boost antitumor CD8^+^ T cell responses and enhance therapeutic efficacy.

Multiple antigenic peptides are good alternatives for peptide presentations. Four or eight copies of peptides were attached to a core comprising lysine residues, thus producing a branched peptide tree with a molecular weight of a small protein [52]. The high concentrations of the repeating peptide sequences and alterations in the three-dimensional structure equipped the peptide with excellent stability and boosted its immunogenicity [53, 54]. Onodi et al. had developed a survivin-based vaccine made up of a pool of three SLPs (SVX) containing six CD8^+^ and eight CD4^+^ T cell epitopes that could bind to various HLA class I and class II molecules [55]. Although survivin is predominantly present in a large proportion of tumor cells, it is usually immune tolerant [56]. The SVX vaccine was demonstrated to activate both CD4^+^ and CD8^+^ immune responses in healthy individuals irrespective of the individual HLA types. In addition, the vaccine was shown to significantly reduce the tumor growth in mice engrafted with colorectal cancer and B-lymphoma, which was associated with the induction of survivin-specific T cell responses. Upon secondary challenge with tumor cells, SVX vaccine was able to generate effective antitumor memory responses which resulted in 100% mice survival up to 60 days [55].

Personalized peptide neoantigen is targeted to stimulate a person's immune system to recognize and kill tumor cells, particularly through delivery of neoantigens to APC, presentation of tumor-specific neoantigen to T cells, and activation of cytotoxic T cells [57]. The tumor antigen-specific T cells infiltrated into the tumor microenvironment can activate the “cold” tumors into “hot” tumors and produce a greater antitumor response. Long-term pancreatic cancer survivors have been found to have significant immunogenic neoantigens and robust CD8^+^ T cell infiltrations, demonstrating that neoantigen-based cancer immunotherapies could prolong survival [58]. A phase I clinical trial in melanoma patients confirmed the efficacy of personalized neoantigen vaccines. Peptide-based neoantigen vaccinations were shown to enhance the regression of melanoma and provided long-term protection against tumor relapse and metastasis [59]. Ott et al. reported that a vaccine of SLPs containing up to 20 predicted neoantigens that were specific to each patient's tumor was feasible, safe, and immunogenic. Activation and expansion of both CD4^+^ and CD8^+^ T cells reactive to multiple neoantigens were observed. Two-thirds of the patients showed clinical responses ranging from no recurrence to a reduction in metastasis [59].

The interaction between the peptide and MHC molecule determines the degree and strength of the immune response, and minor changes in the peptide sequence could have an effect. Thus, various modifications of the peptide sequences were studied. In several investigations, a single amino acid in the peptide sequence was replaced to improve the anchoring of the peptide to MHC groove which boosted the T cell response [43, 60]. Mimotopes or altered peptide ligands are modified peptides that resemble the spatial structure of the presented epitopes rather than their sequences. Although they elicited an increase in T cell numbers than the unchanged peptide, these T cells did not proficiently cross-react and had a lower affinity for the tumor antigen, necessitating additional boosting vaccinations with the native tumor antigen to enhance antitumor immunity [60, 61]. Eventhough this approach enhanced ex vivo CD8^+^ T cell responses in melanoma patients, it did not improve their overall survival [62]. This could be due to the limited number of MHC-peptide complexes exhibited by tumor cells and the lack of expression of costimulatory molecules [60].

## 4. Advantages and Limitations of Peptide-Based Cancer Vaccines

Peptide-based vaccines possess numerous advantages over other therapies. Peptide vaccines have shown benefits in treating metastatic cancers since they lacked significant toxicity associated with chemotherapy and radiotherapy. Unlike chimeric antigen receptor (CAR) T cell immunotherapy which needs to target a cell surface antigen, peptide-based cancer vaccines can use multiple T cell epitopes positioned outside or inside of tumor cells [63]. The risk of hypersensitivity could be avoided, and tumors with high heterogeneity could be effectively targeted by developing a peptide vaccine free from B cell epitopes [64]. Furthermore, peptide-based cancer vaccines can effectively induce significant immune responses for active immunotherapy. Besides, while antigen loss is a significant disadvantage of cellular therapies that rely on a single target antigen, cancer peptide vaccines containing multiple epitopes could address this issue with greater flexibility [63]. Although some severe adverse effects might be correlated with genetic modifications when equipped with high-affinity T cell receptors, genomic alterations of neoantigens including deletions and mutations could elicit endogenous T cell immune reactions in various tumor types [65]. Personal neoantigen vaccine, a new type of peptide-based cancer vaccine, was shown to be safe and efficacious and could robustly elicit T cell responses [66].

Nonetheless, many challenges must be considered when developing peptide-based vaccines. While algorithms for predicting T cell epitopes are extensively used, their accuracy and sensitivity are rather restricted because epitope spatial configuration alters when the antigens bind to cell surface receptors. As a result, false-positive and false-negative results could occur [67]. Eventhough peptides present on MHC-II molecules recognized by helper T cells could significantly enhance the efficacy, it is extremely difficult to predict the immunogenicity of MHC-II-restricted peptides as they are highly diverse and more complex than MHC-I [68]. Since peptide-based vaccines are designed based on different amino acid sequences, product heterogeneity is possible. Thus, in vivo instability could result in an unpredictable biodistribution profile and modulating the therapeutic effects. Developing a peptide-based vaccine for global use is difficult due to MHC restrictions and vast heterogeneity in MHC alleles in the human population [69, 70]. However, the advancement of bioinformatics tools to predict high coverage of HLA alleles enables focusing on the most prevalent MHC alleles and employing promiscuous peptides capable of binding to more than one MHC allele are possible solutions to this problem.

## 5. Role of Adjuvants and Peptide Delivery System

Peptides when given alone as vaccines do not elicit strong immune responses in vivo due to their quick degradation at the injection site, lack of costimulatory ability, and absence of danger signals required for APC activation. Thus, the selection of strong adjuvants or immunostimulators and ideal delivery systems is critical in inducing effective T cell responses as well as ensuring that the peptide vaccine is appropriately sensed by and could activate the immune system. Both adjuvants and delivery systems have the ability to induce an immune response as well as protect the antigen from degradation and deliver it to the desired tissues. Delivery systems are often defined as self-adjuvanting or having a built-in adjuvant that could be administered or transported, while adjuvants are substances with the ability to induce an immune response against the antigen of interest [71]. Adjuvants can be divided into two categories as “depot adjuvants” which prolong antigen availability (e.g., emulsions) and as “immunostimulatory adjuvant” which acts as potentiators of innate and adaptive immune responses (e.g., Toll-like receptor agonist, cytokines, and STING).

### 5.1. Adjuvants

Montanide ISA-51 and Montanide ISA-720 are water-in-oil emulsions that have been widely used as adjuvants because they form a depot at the injection site which prevents the soluble antigens from rapidly trafficking to local lymph nodes, leading to inflammation and the gradual release of the antigens [72–74]. Due to the nonabsorbable mineral oil composition, it could persist at the injection site for weeks to months, assisting in epitope persistence to activate T cells [75]. Combining epitope peptides with Montanide ISA-51 or Montanide ISA-720 could help in eliciting a stronger immune response and destroy more tumor cells. Montanide ISA-51 and ISA-720 were used in cancer vaccines in clinical trials involving different types of cancers such as melanoma and nonsmall cell lung cancer (NSCLC). They were shown to induce antigen-specific antibody and T cell responses that were associated with longer survivals of patients, indicating that Montanide-based adjuvants could be promising adjuvants for cancer vaccines [76, 77]. In addition, Montanide ISA-51 was also reported to elicit both CD4^+^ and CD8^+^ T cell responses in patients who were vaccinated with long peptides of the oncoproteins E6 and E7 [78]. Furthermore, for efficient uptake, the peptides could also be encapsulated in liposomes and nanoparticles or covalently conjugated to adjuvants [79–82].

A major advancement for cancer vaccines is to develop an adjuvant that could target specific immune system components to produce a robust and long-lasting immune response. Adjuvants comprising pathogen-associated molecular pattern molecules (PAMPs) and proinflammatory cytokines are now being used and can provide a danger signal that is recognized by pattern recognition receptors (PRRs) enhancing the immune response. These receptors include the Toll-like receptor (TLR) agonists which are effective adjuvants that could mimic microbial stimulations, and these have shown the ability to enhance epitope-induced CTL memory activation and increased vaccine efficacy in cancers [83, 84]. TLR agonists targeting lymph nodes had demonstrated a direct connection between the magnitude of CD8^+^ T cell responses and the amount of TLR agonists acquired in draining lymph nodes, thus demonstrating the importance of developing adequate inflammatory signals during immunization. Several TLR agonists had been tested as adjuvants for cancer vaccines, and the most commonly used is polyinosinic-polycytidylic acid, a synthetic TLR3 ligand that was stabilized with polylysine and carboxymethylcellulose (poly-ICLC). Such an adjuvant could enhance tumor-specific T cell response by TLR3 signaling, thus preventing T cells from exhaustion and improving immunotherapeutic outcomes [85–88]. Besides, Melssen et al. demonstrated that poly-ICLC could be employed as a successful vaccine adjuvant to induce CD8^+^ T cell immune response with targeted action and tolerable safety in melanoma patients [89].

Cytokines are increasingly being used in cancer immunotherapy, especially in cancer vaccine regimens as they could induce both cellular and humoral immune responses. Cytokines including IFN-*α*, IFN-*γ*, IL-2, IL-12, IL-15, IL-18, and IL-21 exhibited immunological efficacies when they were used as vaccine adjuvants [90]. However, to date, immunostimulatory cytokines such as GM-CSF is the most common adjuvant being used in anticancer peptide vaccination trials as it could enhance effective priming of T cell responses by attracting and stimulating DCs [88, 91, 92]. A peptide-based vaccine utilizing the E75 peptide (HER-2/neu369-377), also known as nelipepimut-S or NeuVax, was coadministered with GM-CSF in phases I and II trials, and production of antigen-specific CD8^+^ T cells was enhanced, leading to progression-free survival in breast cancer patients expressing HLA-A2 and HLA-A3 molecules [93]. Unfortunately, a phase III study failed to show efficacy in preventing breast cancer recurrence. Recombinant GM-CSF has been used in peptide vaccine trials in mice as well as in humans, where it showed various degrees of efficacy in stimulating T cell responses. This might be partially due to a balance between the pro and anti-inflammatory properties of GM-CSF, depending on the dosage. In addition, there might be complex interactions between GM-CSF and other factors in the tumor microenvironment that influenced its ability to either enhance or reduce vaccine-induced T cell responses [94, 95].

Another class of new emerging adjuvants is the stimulator of interferon gene (STING) protein agonist, a transmembrane protein that induces a strong type I IFN response upon activation. STING was expressed at the highest level in T lymphocytes, and STING activation could lead to T cell apoptosis, where such phenomena were not observed with macrophages and DCs [96]. To be used with cancer vaccines, STING agonist would have to be combined with an adjuvant or delivery system that specifically targeted myeloid cells in vivo to prevent T cell apoptosis. Preclinical studies using STING agonists injected directly into tumors in the aggressive B16 melanoma model demonstrated promising results, sparking high potential in their clinical applications [97]. However, limitations such as potential toxicity and lack of specific targeting would have to be overcome.

### 5.2. Delivery Systems as Adjuvants in Vaccine Formulations

The limitations of peptide-based cancer vaccines could be overcome by appropriate formulations. For instance, incorporating drug delivery systems into vaccine formulations could aid in the delivery of peptides to APC. Peptides, as well as adjuvants and targeting sequences, could be encapsulated to enable the delivery of a single package that generates a powerful T cell-mediated response. Poly (lactic-co-glycolic acid) (PLGA) and liposomes are examples of drug delivery applications that have been studied experimentally for many years and had proven history in terms of safety and biodegradability, with the FDA approving their usage as drug delivery systems [98]. PLGA could also act as a self-adjuvant to boost the immune response. Preclinical research had revealed that PLGA nanoparticles could successfully transfer TAAs to APCs, leading to expansion of cytotoxic CD8^+^ T cells and enhanced immunotherapy response [99]. In a recent study, Bae et al. employed PLGA nanoparticles to deliver an immunogenic heteroclitic peptide (BCMA72-80) (YLMFLLRKI) encapsulated in PLGA to enhance antigen delivery and presentation. More robust BCMA-specific CD8^+^ CTL responses were achieved against multiple myeloma rather than vaccination with the free peptide. Thus, the results demonstrate the potential clinical application of PLGA-based cancer vaccines to enhance BCMA-targeted immunotherapy against multiple myeloma [100].

In addition, the surface of PLGA nanoparticles could be modified with DC-specific antibodies such as anti-CD11c and anti-DEC-205, which targeted elements such as mannose or TLR. The modified PLGA nanoparticles had stronger interactions with DC receptors, leading to higher uptake of nanoparticles and DC maturation [101, 102]. Many studies had shown codelivery of cancer antigens and adjuvants to the same DC improved DC activation, antigen presentation, and therapeutic T cell response [101, 103]. A PLGA nanoparticle-based vaccine was generated for codelivery of MUC-1 peptide BLP25 together with MPLA [104]. This formulation had effectively activated naive T cells of normal and MUC-1 transgenic mice. These findings suggested that PLGA nanoparticles could be used to deliver vaccines to DCs effectively. Besides, by encapsulating murine melanoma antigenic peptides (hgp10025-33 and TRP2180-188) and MPLA in PLGA nanoparticles, stronger antigen-specific immune responses were produced compared to the use of Freund's adjuvant. Vaccination with this nanoparticle-mediated peptide was shown to significantly delay the growth of subcutaneously inoculated B16 melanoma cells in murine models [105]. Hamdy et al. had coencapsulated tyrosinase-related protein 2 (TRP2180-188) and TLR ligand 7-acyl lipid A into PLGA nanoparticles. The vaccine was able to significantly enhance therapeutic antitumor effects in B16 melanoma tumors. The activated TRP2-specific CD8 T cells were proficient to secrete IFN-*γ* in the lymph nodes and spleens of vaccinated mice [106]. The findings suggested that PLGA nanoparticles could be potentially used as effective carriers in the development of future peptide-based cancer vaccines.

Liposomes are phospholipid bilayers that resemble cell membranes and are extremely customizable. Liposomes could be tailored in terms of size, charge, surface properties, and delivery mechanism. These characteristics allowed liposomes to mimic the size of pathogens and surface markers [107, 108]. Liposomes offered peptides better access to the spleen and lymph nodes which have higher proportions of cross-presenting DCs, and the particulate systems could protect the peptides from degradation and release [109]. Following internalization, the liposomes continued to enhance antigen cross-presentations by allowing its peptide load to escape from the lysosome and into the cytosol, which is an important step in antigen cross-presentation and stimulation of a robust CD8^+^ T cell response [110]. An example of a liposome-based delivery system was reported by Rueda et al. where the nanoliposomes encapsulated multiantigenic T helper cell epitopes targeting LHR hormones, tetanus toxin immunogen as adjuvants, and external Fc receptor ligands which enhanced liposome uptake by DCs [111]. Cationic liposomes were shown to work better than PLGA-NP for delivering long peptides and inducing cell-mediated immunity. Varypataki et al. reported that cationic liposomes could work more efficiently than PLGA-NPs to deliver long peptides as it induced the highest in vivo killing activity [112].

In another study, Arab et al. developed an effective vaccine delivery system by incorporating the epitope E7, derived from the highly expressed antigen HER-2 in breast cancer patients to the surface of liposomes containing distearoyl phosphatidylcholine (DSPC) and distearoyl phosphatidylglycerol (DSPG) to enhance antitumor activity. The antigen-specific IFN-*γ* response of CD8^+^ T cells was found to be greatly improved, and CTL antitumor responses were elicited in vaccinated mice. Enhanced CTL responses by this formulation led to the inhibition of tumor growth and prolonged survival time in mice. The findings suggested that liposomes containing DSPC/DSPG/Chol/DOPE could be excellent candidates for the E75 peptide vaccine in the prevention and treatment of HER-2-positive breast cancer [113].

A liposome-based codelivery system containing melanoma-associated antigen-derived peptide GP100280-288 and TLR4 ligand monophosphoryl lipid A (MPLA) had been developed which could be phagocytized by subcutaneous DCs and significantly enhanced the epitope-specific T cell response. These findings suggested that the approach of using nanocarriers based on liposomes was effective to elicit antitumor immune responses [114]. In addition, Zamani et al. designed a nanoliposomal vaccine consisting of a P5 peptide, a CTL-specific peptide derivative of the rat HER-2/neu protein, a Pan HLA-DR epitope (PADRE) peptide as well as MPLA (a toll-like receptor 4 ligands) [115]. PADRE is a universal HLA-DR-restricted CD4^+^ T helper cell epitope that elicited a CD4^+^ T cell response in the majority of patients. By using DOPE in the liposome design, the nanoparticle formed a hexagonal structure at low pH and permitted the particle to fuse with the endosomal wall, resulting in an escape into the cytosolic pathway for MHC class I cross-presentation [115, 116]. It was found that the combination of liposome-P5 peptide integrated with PADRE-MPL formulation significantly increased the production of IFN-*γ* and CD^+^8 T cells. Tumor growth was reduced, and improved survival was observed compared to other groups of treated mice [115]. Another study using a different HER-2/neu-derived peptide demonstrated similar findings and showed enhanced CD4+ and CD8+ T cell responses as well as enhancement of IFN-*γ* production [117]. These findings revealed that liposomal formulations containing long multiepitope peptide E75-AE36 with PADRE could be used as an effective multiepitope prophylactic/therapeutic vaccine to generate potent antigen-specific CD8^+^ T cell immune responses.

Currently, there has been no consensus regarding the most optimal adjuvant to be used for a given peptide vaccine, and this could be a promising research area to further optimize and improve vaccines formulations [118]. All currently active or recruiting phases I and II peptide-based cancer vaccine trials in combination with immunological adjuvants including Montanide ISA-51, poly-ICIC, GM-CSF, and others are given in Table 3.

## 6. Combinatorial Therapy of Peptide-Based Cancer Vaccines

The tumor microenvironment remains a challenging obstacle for highly functional tumor-specific T cells induced by peptide vaccines. Tumor cells, myeloid cells, regulatory T (Treg) cells, and abnormal vasculature could lead to the suppression of T cell infiltrations or functions. Effective immune therapy might entail the induction of T cell responses to multiple antigens at the same time and the maintenance of T cell activations in tumor mass. Effective control of increased tumor load requires multiple combinations of therapeutic strategies. The increase in clinical cancer immunotherapy provides a wide array of prospects for rational immunotherapy combinations with peptide vaccinations. Many of the immunotherapeutic approaches are already in clinical trials, and these include immune checkpoint inhibitors and neutralizing antibodies to inhibitory cytokines. Synergistic combinations are not only restricted to immunotherapy but can be combined with radiotherapy and chemotherapy.

### 6.1. Peptide-Based Cancer Vaccines with Other Treatment

Combinations of peptide-based cancer vaccines with conventional anticancer treatment are prevalent as patients are generally treated with radiotherapy, chemotherapy, and immunotherapy as part of regular care practices. For instance, radiotherapy might have a partial effect on some tumor lesions. The treatment might not reach all tumor targets that have metastasized or tumors of large sizes. Phase I clinical study for advanced hepatocellular carcinoma had shown that tumors could be effectively prevented by combining personalized peptide vaccine with radiotherapy. The liver masses were shown to reduce significantly in size within and outside of the radiation area after the combination treatment [119].

Cyclophosphamide is a chemotherapeutic drug that has direct cytotoxicity at high dosages but demonstrated immunomodulatory effects at low dosages as suppression of Treg cells and enhanced IFN-*γ*^+^ tumor-specific T cell responses were able to delay tumor progression [120]. Thus, the combination of low-dose cyclophosphamide with peptide-based cancer vaccines could provide clinical benefits since cyclophosphamide could selectively deplete Tregs and modulate dendritic cell homeostasis [121, 122]. A phase I clinical trial of RNF43 peptide-pulsed DCs combined with low-dose cyclophosphamide and IL-2 was shown to be safe. The combinations reduced the frequency of peripheral blood Tregs, leading to a good clinical response in patients with RNF43-positive advanced solid tumors [121]. In an open-label randomized phase II trial, the combinatorial treatment of personalized peptide vaccine (PPV) with cyclophosphamide was reported to provide clinical benefits in advanced biliary tract cancer (aBTC) patients. The T cell responses to the peptides used in vaccination were generally higher in the PPV/cyclophosphamide arm than in the PPV alone arm. The PPV/cyclophosphamide arm also showed significantly improved progression-free survival and overall survival when compared to the PPV alone arm. After immunizations, the PPV alone arm had a significant induction of plasma IL-6, but not the PPV/cyclophosphamide arm, which could be linked to the suppression of antigen-specific T cell responses. These findings suggested that combined treatment of low-dose cyclophosphamide in aBTC patients with PPV would provide clinical advantages, presumably by preventing IL-6-mediated immune suppression. More clinical trials are required to evaluate the clinical efficacy of PPV/cyclophosphamide in aBTC patients [122].

Gemcitabine is an anticancer chemotherapy drug that has immune-modulating properties such as enhancing antigen cross-presentation as well as inhibiting myeloid-derived suppressor cells and Treg cells [123, 124]. Gemcitabine was shown to enhance the expression of the Wilms tumor gene 1 (WT1) and induced the sensitivity of pancreatic cancer cells to WT1-specific T cell-mediated antitumor immune response [125]. Phase I clinical trial reported that the efficacy of combination treatment of WT1 peptide-based vaccine with gemcitabine was more efficacious compared to treatment with gemcitabine alone [126]. In a phase II randomized study, the combination of gemcitabine with WT1 peptide vaccination was found to significantly prolong progression-free survival and overall survival in patients with advanced pancreatic ductal adenocarcinoma. The combination showed reduced tumor burden and long-term disease stability without unexpected toxicities [127].

Trastuzumab, an anti-HER-2 monoclonal antibody, was used to treat breast cancer and had been found to cause HER-2-positive tumor cells to become more susceptible to antibody-dependent and T cell-mediated cytotoxicity [128, 129]. In vitro and in vivo studies by Gall et al. showed that trastuzumab enhanced the DC uptake and cross-presentation of HER-2-derived peptides (E75), leading to antitumor immune priming and enhanced production of antigen-specific CTLs [130]. In addition, the combination of trastuzumab with GM-CSF and E75 peptide (nelipepimut-S) in a phase IIb clinical trial was shown to be safe with no added toxicity compared to trastuzumab alone even after prolonged exposure. No significant difference in disease-free survival was observed in HER-2 low-expressing breast cancer, but the significant clinical benefit was seen in patients with triple-negative breast cancer (TNBC) [131, 132]. These findings suggested that a combination of nelipepimut-S and trastuzumab could be used as adjuvant therapy for early TNBC and warrant additional studies in phase III randomized trials.

Low-dose dexamethasone is one of the alternative therapies for castration-resistant prostate cancer (CRPC), either alone or in combination with PPV [133–135]. The combination of PPV plus low-dose dexamethasone was shown to provide clinical benefits in a randomized phase II trial of CRPC patients. The overall survival was significantly longer when compared to dexamethasone alone (73.9 vs. 34.9 months; *p*=0.00084) due to induction of the specific antitumor immunity [136]. In another study, Noguchi et al. examined the antitumor effects of a mixture of 20 peptides with docetaxel and dexamethasone in patients with CRPC [137]. The combination resulted in decreased prostate-specific antigen levels, induced immune response, and reduced immunosuppressive myeloid-derived suppressor cells. However, the combination failed to demonstrate a strong synergistic efficacy with no enhancement in the overall survival [137]. Additional large-scale clinical trials comparing the overall survival are needed to establish the clinical benefits of the treatment.

### 6.2. Peptide-Based Cancer Vaccines and Immune Checkpoint Inhibitor

**Figure 1:**
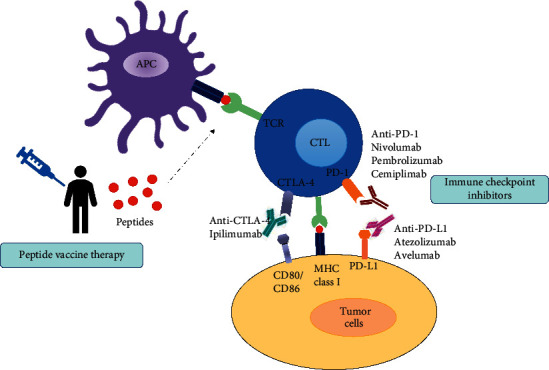
Mechanism of action of immune checkpoint inhibitor therapy and peptide vaccine therapy. Injected peptides induce peptide-specific cytotoxic T lymphocyte through antigen-presenting cells leading to antitumor effects on cancer cells. Immune checkpoint therapy could result in antitumor effects by inhibiting the mechanism that negatively suppresses the immune response to tumor cells. Checkpoint blockade with monoclonal antibodies against PD-1, PD-L1, and CTLA-4 could block the interaction of these receptors or ligands from binding to their partners, resulting in sustained T cell activation antitumor responses.

Peptide-based cancer vaccines have yet to demonstrate efficacy in the clinic as a monotherapy. Nevertheless, research has demonstrated that peptide-based cancer vaccines could be used in combination with other immunotherapies to improve potency over single-agent therapy. Combining peptide-based cancer vaccines with an immune checkpoint inhibitor (ICI) is one such example. The development of ICIs is a milestone in cancer immunotherapy that results in anticancer effects by blocking the mechanisms that suppress the immune response to tumor cells (Figure 1). ICIs have been proven to enhance the existing antitumor immune response by targeting the immune checkpoints and promoting immune-mediated eradication of tumor cells [138]. Immune checkpoints are increasingly expressed by effector T cells in the tumor microenvironment as cancer progress, resulting in a decreased capacity to kill tumor cells. The phenomenon known as “T cell exhaustion” could be restored by using ICIs, which are now used to treat many types of cancer [139]. Currently, several monoclonal antibodies were being developed targeting PD-1 (nivolumab, cemiplimab, and pembrolizumab), PD-L1 (atezolizumab, durvalumab, and avelumab), and CTLA-4 (ipilimumab) [140].

To examine the efficacy of combination therapies, many researchers combined their treatments with ICIs and other anticancer drugs. For example, two phase I trials showed that nivolumab, the antiprogrammed death-1 (PD-1) antibody, when used in combination with a multipeptide-based vaccine targeting differentiation antigens was well tolerated, safe, and produced immune responses in melanoma patients [141, 142]. In the trial, patients with ipilimumab-refractory or naive melanoma (*n* = 90) were treated with multiple doses of nivolumab with or without multipeptide vaccine (gp100, MART-1, and NY-ESO-1 with Montanide ISA-51 VG) (NCT01176461). In both groups of patients with ipilimumab-refractory or naive melanoma, the treatment with nivolumab and peptide vaccination was well tolerated and induced durable response up to 140 weeks. However, the inclusion of the peptide vaccine did not improve the clinical efficacy of the PD-1 blockade [141]. In another phase I trial, a combination of nivolumab with the multipeptide vaccine (gp100, MART-1, and NY-ESO-1 with Montanide ISA-51 VG) as an adjuvant in resected stages IIIC and IV patients with metastatic melanoma was conducted by the same group. Both relapse-free survival and overall survival were promising compared with the previous trial. The median overall survival in the study was not reached with a median follow-up period of 32.1 months, and the recurrence rate was significantly reduced to 30.3%. This study indicated that combining nivolumab with the multipeptide-based vaccine might enhance immunologic response and improve survival rates in high-risk resected melanoma patients [142].

In addition, a combination of nivolumab and ISA101 HPV-16 synthetic long peptide vaccine in phase II clinical trial (NCT02426892) also showed positive results and provided evidence of the effectiveness of the combinations to enhance the efficacy of vaccine-activated T cells in the immunosuppressive tumor environment. The combined vaccination showed promising results in 24 patients with an overall response rate of 33% with a median overall survival of 17.5 months when compared to 20% overall response rate and median survival of approximately 9 months with nivolumab alone in similar patients with advanced cervical cancer [143]. The ISA101 vaccine is now being tested in combination with cemiplimab (anti-PD-1 antibody, NCT03669718) and utomilumab (anti-4-1BB antibody, NCT03258008). Different studies evaluated a personal neoantigen vaccine (NEO-PV-01) in combination with nivolumab in patients with advanced melanoma, NSCLC, or bladder cancer in a phase 1b clinical trial (NCT02897765) [144]. The combinatorial therapy was demonstrated to be safe and well tolerated with no treatment-related serious adverse events. All of the patients showed neoantigen-specific CD4^+^ and CD8^+^ T cell responses after vaccination using an ex vivo assay with peripheral blood samples. The T cells induced by the vaccine had a cytotoxic phenotype which was proficient in trafficking to the tumor and killing the cells.

Recently, the study by Tanaka et al. utilizing a novel multiepitope long peptide vaccine, TAS0314, demonstrated a synergistic antitumor immunity in combination treatment with PD-1/PD-L1 blockade in HLA-A*∗*2402 knock-in mice [145]. The combination of TAS0314 plus anti-PD-1 antibody dramatically reduced tumor development and prolonged survival when compared to monotherapy alone. An increase in the number of epitope-specific CTLs by three-fold was reported, and this increase indicated the mechanism underlying the synergistic anticancer impact of TAS0314 treatment with PD-1 and PD-L1 antibodies. Liu et al. demonstrated that their novel synthetic vaccine, scFv(DEC-205) and HPV-16 E7 long peptide (scFv(DEC-205)-E7), produced a significant therapeutic antitumor response in TC-1 tumor-bearing mice. Interestingly, combinational therapy with PD-L1 blockade further improved the antitumor effect of the scFv(DEC-205)-E7 vaccine [146].

The efficacy and safety of combined treatment of ipilimumab, a monoclonal anti-CTLA-4 antibody with peptide vaccination (gp100), were investigated in patients with metastatic melanoma. The clinical response data demonstrated a durable objective response which was accompanied by autoimmunity and cancer regression [147]. This promising result was not reproducible in phase III trials (NCT00094653), a combination of ipilimumab plus gp100 emulsified in incomplete Freund's adjuvant (IFA). The study reported no difference in median overall survival between the combined treatment and ipilimumab alone (10.0 vs. 10.1 months). The adverse events might be durable, severe, or both, but most can be reversed with appropriate treatment. Overall, their findings showed that ipilimumab with peptide vaccination (gp100) did not improve clinical outcomes [148]. According to Hailemichael et al., IFA exerts a formation of depot at the site of vaccination, which might sequester antigen-specific T cells leading to inhibition of T cell migration to the tumor site [149, 150]. This may explain why the gp100 vaccine was unsuccessful (with IFA adjuvant).

Recently, data from the phase I/IIa trial evaluating UV1 vaccinations with GM-CSF as an adjuvant in combination with ipilimumab showed a positive impact on patients with metastatic melanoma (NCT02275416) [151]. UV1 is a therapeutic cancer vaccine that consisted of three synthetic long peptides of the enzyme telomerase (hTERT) which could induce CD4^+^ T helper type 1 (Th1) cells. The combination treatment was well tolerated and induced a clinical response in melanoma. Ten out of 11 patients (91%) showed Th1 immune response against UV1 peptides in pre and postvaccinations, suggesting the synergistic effects between UV1 vaccine with ipilimumab. The overall survival was 50% at 5 years, providing encouraging signals of long-term survival benefits for UV1 in this late-stage patient population and when compared to previous data of ipilimumab monotherapy. Findings from this study support the use of UV1 in combination with ipilimumab and nivolumab, which is currently used as a first-line treatment for advanced melanoma. A phase I trial investigating combination treatment of UV1 with pembrolizumab (NCT03538314) and a phase II trial of UV1 vaccination with nivolumab and ipilimumab (NCT04382664) are currently fully recruited and ongoing, respectively.

Despite the lack of clinical data on the combination of ICI with peptide-based vaccines, various phase I or II clinical trials are currently active and/or recruiting.  Table 4 provides the ongoing trials combining ICI with peptide-based vaccines with or without conventional therapies (radio and chemotherapy).

## 7. Conclusion

Peptide-based vaccines are attractive immunotherapeutic options as they are safe and inexpensive to produce. It can elicit cell-mediated antitumor response via antigen presentation of selected tumor epitopes to T cells. Peptides are also promising since they are highly specific to elicit antigen-specific T cell response and could be employed in multiplexing strategies targeting multiple epitopes. Since peptide-specific immunity has been shown to decrease over time, peptide-based vaccines targeting different tumor antigens need to overcome resistance. Therefore, the ideal peptide vaccine should include multiple peptides derived from different antigens to boost CD4^+^ with CD8^+^ T cell response as well as mutated and unmutated tumor-associated peptides. New approaches are enabling the identification and development of more immunogenic TAAs and TSAs. Although some peptide-based cancer vaccines have demonstrated improved survivals with fewer side effects when compared to conventional therapies, this treatment as monotherapy is deemed insufficient to achieve long-term cancer control and cure. Peptide-based cancer vaccines are currently being tested on a wide range of targets, demonstrating their versatility. There is a notable direction in most of the studies towards a more personalized approach to the selection of patient neoepitopes and most often in combinations with immunological adjuvants such as Montanide ISA-51, poly-ICLC, and GM-CSF. Peptide-based cancer vaccines have also shown an increased focus in combinations with standard treatment regimens such as chemotherapy, radiotherapy, or various immunotherapeutic approaches. The combination of peptide-based anticancer vaccines with immunotherapies such as immune checkpoint blockade that enhance T cell responses and the presentation of tumor-associated antigens in immunopeptidome could lead to the induction of stronger antitumor responses. Combinations with checkpoint modulators and other novel drugs that reverse immunosuppression are rapidly improving, although more research studies are needed to establish the effectiveness of combination therapies and which combinations are the best, as well as the optimum dose scheduling for each component.

## Figures and Tables

**Table 1:** FDA-approved prophylactic and therapeutic cancer vaccines.

Cancer vaccine	Strategy	Associated cancer	Name of the vaccine	Indication/study details
Prophylactic	Viral antigen-based vaccines	HPV-related anal, cervical, head and neck, penile, vulvar, and vaginal cancers	Cervarix	(1) Approved for use in females aged 9 through 25 years.
				(2) By intramuscular injection and consist of 3 doses (0.5 ml each) at 0, 1, and 6 months
			Gardasil	(1) HPV quadrivalent recombinant vaccine (types 6, 11, 16, and 18)
				(2) Approved for use in females and males aged 9 through 26 years
			Gardasil-9	(1) HPV-9 valent vaccine (recombinant)
				(2) Approved for use in females and males from 9 to 45 years of age
		HBV-related hepatocellular carcinoma	Engerix-B	(1) Hepatitis B vaccine (recombinant)
				(1) Prevention against infection caused by all known subtypes of hepatitis B virus.
				(2) Administer intramuscularly two doses (0.5 ml each) separated by one month
			Recombivax HB	(1) Hepatitis B vaccine (recombinant)
				(2) Approved for use in adult predialysis and dialysis patients aged 18 years and older
			Heplisav-B	(1) Hepatitis B vaccine (recombinant), adjuvanted
				(2) Approved for use in adults aged 18 years and older
Therapeutic	Attenuated bacteria	Early stage bladder cancer	Bacillus Calmette-Guérin (BCG)	(1) For the treatment and prophylaxis of carcinoma in situ (CIS) of the urinary bladder.
				(2) For the prophylaxis of primary or recurrent state Ta and/or T1 papillary tumors following transurethral resection
	Cell-based vaccines	Metastatic castration-resistant prostate cancer	Sipuleucel-T (Provenge)	(1) Autologous cellular immunotherapy
				(2) For asymptomatic or minimally symptomatic prostate cancer with metastases that are resistant to standard hormone treatment
				(3) Administered intravenously in a three-dose schedule at two-week intervals
	Oncolytic virotherapy	Advanced melanoma	Talimogene laherparepvec; T-VEC (IMLYGIC)	(1) Genetically modified HSV that expressed GM-CSF
				(2) Durable response rate (DRR) (16.3%) was shown in patients with unresected stage IIIB to stage IV melanoma administered with T-VEC when compared to GM-CSF (2.1%)

**Table 2:** Different types of tumor antigens.

	Class of tumor antigen	Description	Tumor specificity	Example of tumor antigen	References
Tumor-associated antigens (TAA)	Overexpressed antigens	Antigens overexpressed in tumor cells and normal level of expression in healthy cells	Variable	HER-2, hTERT, mesothelin, MUC-1, and p53	[16–19]
	Differentiation antigens	Antigens expressed on tumor cells and normal cells	Variable	gp100, MART-1, PSA, PAP, and tyrosinase	[20–24]
	Cancer-testis antigens	Antigens are primarily expressed on testes, fetal ovaries, and trophoblasts	High	BAGE, MAGE, GAGE, PRAME, NY-ESO-1	[25–28]
Tumor-specific antigens (TSA)	Oncogenic viral antigens	Abnormal expression in cells infected with an oncovirus	High	EBV LMP-1/LMP-2A, HPV-E6/E7, HTLV-1	[29–32]
	Tumor-specific mutated antigens	Mutations result in the generation of a new peptide. Mutations could arise at the gene level from chromosome translocations or due to posttranslational modifications	High	KRAS, NRAS, epitopes from BCR-ABL translocation, ETV6, NPM/ALK and ALK	[33]

HER-2, human epidermal growth factor receptor 2; hTERT, human telomerase reverse transcriptase; MUC-1, mucin 1; MART-1, melanoma antigen recognized by T cells; PSA, prostate-specific antigen; PAP, prostatic acid phosphatase, MAGE, melanoma antigen; NY-ESO-1, New York esophageal squamous cell carcinoma 1; GAGE, G antigen; BAGE, B melanoma antigen; EBV, Epstein–Barr virus; LMP-1, latent membrane protein 1; LMP-2A, latent membrane protein 2A; HPV, human papillomavirus; HTLV-1, human T cell lymphotropic virus type 1; KRAS, Kirsten rat sarcoma virus; NRAS, neuroblastoma RAS viral oncogene homolog; BCR, breakpoint cluster region gene; ABL, Abelson proto-oncogene; NPM, nucleophosmin; ALK, anaplastic lymphoma kinase.

**Table 3:** Phase I and phase II clinical trials of peptide-based therapeutic cancer vaccines in combination with immunological adjuvants currently active or recruiting.

Cancer types	Peptide vaccine	Adjuvants	Phase	Recruitment status	Clinical trials
Breast cancer	ESR1	Montanide ISA and GM-CSF	I	Recruiting (2020–2024)	NCT04270149
	HER-2	GM-CSF	I	Recruiting (2019–2023)	NCT04144023
Colorectal cancer	Multiple peptide PolyPEPI1018 vaccine	Montanide	II	Completed (2018-2019)	NCT03391232

Glioblastoma	Multipeptide and the immune modulator XS15	Montanide ISA-51	I	Recruiting (2021–2024)	NCT04842513
	Telomerase-derived helper peptides (UCPVax)	Montanide ISA-51	II	Recruiting (2020–2023)	NCT04280848

Leukemia	Personalized peptide vaccine	TLR1/2 ligand XS15	I	Recruiting (2020–2024)	NCT04688385
	PD-L1 and PD-L2 peptides	Montanide ISA-51	II	Active, not recruiting (2019–2021)	NCT03939234
	Personalized peptide vaccine	GM-CSF and imiquimod	II	Active, not recruiting (2018–2021)	NCT03559413

Melanoma	Mutated neoantigen peptide (BRAF/CD4 epitopes)	CD40 antibody and poly-ICLC	II	Recruiting (2020–2025)	NCT04364230
	NY-ESO-1 cancer-testis antigen	Encapsulated in PLGA nanoparticle	I	Recruiting (2021-2022)	NCT04751786
	Arginase-1 peptide	Montanide ISA-51	I	Recruiting (2018–2021)	NCT03689192
	Personalized peptide vaccine	CAF09b	II	Recruiting (2018–2022)	NCT03715985
Myeloma	PD-L1	Montanide ISA-51	II	Recruiting (2019–2021)	NCT03850522

Pancreatic cancer	KRAS	Poly-ICLC	I	Not yet recruiting (2021–2025)	NCT05013216
	Neoantigen peptide	Poly-ICLC	I	Recruiting (2019–2023)	NCT03956056

Prostate cancer	PGV001 (multiple peptide)	CDX-301	I	Recruiting (2021–2031)	NCT05010200
	Bcl-xL	CAF09b	I	Recruiting (2018–2021)	NCT03412786
	RV001V	Montanide ISA-51	II	Active, not recruiting (2019–2022)	NCT04114825

**Table 4:** Phase I and phase II clinical trials of peptide-based cancer vaccines with checkpoint inhibitors with or without conventional therapies that are currently active or recruiting.

Cancer types	Vaccine formulation	Combination	Phase	Recruitment status	Clinical trials
Breast cancer	AE37	Pembrolizumab	II	Active, not recruiting (2019–2024)	NCT04024800
	Multipeptide cancer vaccine (PVX-410)	Pembrolizumab, chemotherapy	II	Not yet recruiting (2020–2025)	NCT04634747
Gastric cancer	Multiple peptide (OTSGC-A24)	Nivolumab, ipilimumab	I	Recruiting (2018–2024)	NCT03784040
Glioma	IDH1R132H peptide	Avelumab	I	Recruiting (2019–2022)	NCT03893903
Glioblastoma	Novel multipeptide (EO2401)	Nivolumab, nivolumab/bevacizumab	II	Recruiting (2019–2023)	NCT04116658
Liver cancer	DNAJB1-PRKACA peptide	Nivolumab, ipilimumab	I	Recruiting (2020–2024)	NCT04248569

Melanoma	Personalized neoantigen peptides (NeoVax)	Nivolumab, CDX-301	I	Not yet recruiting (2021–2027)	NCT04930783
	Personalized neoantigen vaccine (NeoVax) + poly-ICLC + Montanide	Nivolumab, ipilimumab	I	Recruiting (2019–2026)	NCT03929029
	UV1 vaccine + GM-CSF	Pembrolizumab	I	Active, not recruiting (2018–2022)	NCT03538314
	UV1 vaccine	Nivolumab, ipilimumab	II	Recruiting (2020–2024)	NCT04382664
	Neoantigen peptides + rhGM-CSF + anti-PD-1+imiquimod	Toripalimab (anti-PD-1)	I	Recruiting (2019–2022)	NCT04072900

Multiple cancers and solid tumors	KRAS + poly-ICLC	Nivolumab, ipilimumab	I	Recruiting (2019–2024)	NCT04117087
	Personalized peptide vaccine (PANDA-VAC) + poly-ICLC	Pembrolizumab	I	Not yet recruiting (2021–2032)	NCT04266730
	GRT-C903 and GRT-R904 peptide	Nivolumab, ipilimumab	I/II	Recruiting (2019–2023)	NCT03953235
	Personalized adjuvanted vaccine GEN-009 (synthetic long peptides)	Nivolumab, pembrolizumab	I/II	Active, not recruiting (2018–2022)	NCT03633110
	IDO and PD-L1 peptides (IO102-IO103)	Pembrolizumab	II	Not yet recruiting (2021–2024)	NCT05077709
	Telomerase-derived helper peptides (UCPVax)	Atezolizumab	II	Recruiting (2019–2022)	NCT03946358

Nonsmall cell lung cancer	Multiple peptide (GRN-1201)	Pembrolizumab	II	Recruiting (2018–2023)	NCT03417882
	Telomerase-derived helper peptides (UCPVax)	Nivolumab	II	Recruiting (2020–2025)	NCT04263051

Ovarian cancer	Personalized neoantigen (NeoVax) + poly-ICLC	Nivolumab	I	Recruiting (2019–2028)	NCT04024878
	OSE2101 + Montanide	Pembrolizumab	II	Recruiting (2021–2025)	NCT04713514
Pancreatic cancer	Personalized peptide vaccine (PEP-DC vaccine)	Nivolumab, gemcitabine, capecitabine	I	Recruiting (2018–2028)	NCT04627246
